# Computer-aided diagnosis for optical diagnosis of diminutive colorectal polyps including sessile serrated lesions: a real-time comparison with screening endoscopists

**DOI:** 10.1055/a-2009-3990

**Published:** 2023-03-02

**Authors:** Britt B. S. L. Houwen, Yark Hazewinkel, Ioannis Giotis, Jasper L. A. Vleugels, Nahid S. Mostafavi, Paul van Putten, Paul Fockens, Evelien Dekker, POLAR Study Group, B. A. Bastiaansen, K. M.A.J. Tytgat, M. van der Vlugt, H. Beaumont, D. Ramsoekh, P. van Boeckel, N. van Lelyveld, J. Tenthof van Noorden, K. Boparai, T. Kuipers, L. Noach, E. van der Zanden, F. ter Borg, P. van Oosterwijk, R. de Vries, S. Carballal, M. Daca, I. Ordas, L. Moreira, M. Pellisé, M. Cazemier, J. M. Jansen, B. van Eijk, E. J. van Soest, A. Vehmeijer, V. Koussoulas, W. Marsman, F. J Rando Muños, R. Schröder, F. Voogd, B. Zuur

**Affiliations:** 1 Department of Gastroenterology and Hepatology, Amsterdam University Medical Center, Amsterdam, the Netherlands; 2 Department of Gastroenterology and Hepatology, Radboud University Nijmegen Medical Center, Radboud University of Nijmegen, Nijmegen, The Netherlands; 3 ZiuZ Visual Intelligence, Gorredijk, the Netherlands; 4 Department of Gastroenterology and Hepatology, Subdivision Statistics, Amsterdam University Medical Center, Academic Medical Center, University of Amsterdam, Amsterdam, the Netherlands; 5 Department of Gastroenterology and Hepatology, Medical Center Leeuwarden, Leeuwarden, The Netherlands; 6 Bergman Clinics Maag and Darm Amsterdam, Amsterdam, The Netherlands; 7 Amsterdam University Medical Centres and Bergman Clinics Maag & Darm Amsterdam, Amsterdam; 8 Amsterdam University Medical Centres, Amsterdam, Bergman Clinics Maag & Darm Amsterdam, Amsterdam, and Amstelland Hospital, Amstelveen; 9 Sint Antonius Ziekenhuis, Nieuwegein; 10 Amstelland Hospital, Amstelveen; 11 Deventer Hospital, Deventer; 12 Onze Lieve Vrouwe Gasthuis, Amsterdam and Hospital Clinic of Barcelona, Barcelona; 13 IDIBAPS, Barcelona; 14 Spaarne Ziekenhuis, Hoofddorp; 15 Nij Smellinghe Hospital, Drachten); N. van Lelyveld (Sint Antonius Ziekenhuis, Nieuwegein; 16 Medical Center Leeuwarden, Leeuwarden; 17 Tjongerschans Hospital, Heerenveen

## Abstract

**Background** We aimed to compare the accuracy of the optical diagnosis of diminutive colorectal polyps, including sessile serrated lesions (SSLs), between a computer-aided diagnosis (CADx) system and endoscopists during real-time colonoscopy.

**Methods** We developed the POLyp Artificial Recognition (POLAR) system, which was capable of performing real-time characterization of diminutive colorectal polyps. For pretraining, the Microsoft-COCO dataset with over 300 000 nonpolyp object images was used. For training, eight hospitals prospectively collected 2637 annotated images from 1339 polyps (i. e. publicly available online POLAR database). For clinical validation, POLAR was tested during colonoscopy in patients with a positive fecal immunochemical test (FIT), and compared with the performance of 20 endoscopists from eight hospitals. Endoscopists were blinded to the POLAR output. Primary outcome was the comparison of accuracy of the optical diagnosis of diminutive colorectal polyps between POLAR and endoscopists (neoplastic [adenomas and SSLs] versus non-neoplastic [hyperplastic polyps]). Histopathology served as the reference standard.

**Results** During clinical validation, 423 diminutive polyps detected in 194 FIT-positive individuals were included for analysis (300 adenomas, 41 SSLs, 82 hyperplastic polyps). POLAR distinguished neoplastic from non-neoplastic lesions with 79 % accuracy, 89 % sensitivity, and 38 % specificity. The endoscopists achieved 83 % accuracy, 92 % sensitivity, and 44 % specificity. The optical diagnosis accuracy between POLAR and endoscopists was not significantly different ( *P* = 0.10). The proportion of polyps in which POLAR was able to provide an optical diagnosis was 98 % (i. e. success rate).

**Conclusions** We developed a CADx system that differentiated neoplastic from non-neoplastic diminutive polyps during endoscopy, with an accuracy comparable to that of screening endoscopists and near-perfect success rate.

## Introduction

Endoscopic characterization of diminutive (1–5 mm) polyp histology during real-time colonoscopy, so-called optical diagnosis, remains the most attractive intervention for an immediate cost saving in screening colonoscopy 1 . With accurate optical diagnosis, diminutive lesions can be resected and discarded without pathological assessment, or left in place without resection in cases of diminutive non-neoplastic polyps located in the distal colon 2 . Unfortunately, this approach has not yet become a reality. Endoscopists fear the clinical and legal consequences of an incorrect optical diagnosis. In addition, despite the availability of optical diagnosis classification methods, the performance of optical diagnosis by endoscopists varies greatly and not all endoscopists are able to meet the competence thresholds for adoption of optical diagnosis in clinical practice (i. e. the Simple Optical Diagnosis Accuracy [SODA] standards set by the European Society of Gastrointestinal Endoscopy 3 , and the Preservation and Incorporation of Valuable endoscopic Innovations [PIVI] standards set by the American Society for Gastrointestinal Endoscopy 4 ).

With the recent improvements in machine-learning techniques, computer-aided diagnosis (CADx) systems have been developed to improve the accuracy and reliability of endoscopists’ optical diagnosis 5 . These advances have already led to the introduction of the first commercially available, regulatory-approved CADx systems 6 . Although several of these CADx systems were able to reach the PIVI and SODA thresholds within prospective validation settings, none of the systems have yet been able to reach all performance thresholds 7 8 9 10 11 . In addition, there remain several limitations that might hamper widespread implementation of these systems in clinical practice 12 . First, the generalizability and reliability of the results of prospective clinical trials are still limited. This is related to the fact that most systems are tested in single-center studies, and/or are not tested in a real-time endoscopy setting or compared with the performance of a representative group of endoscopists. Additionally, a CADx system should not omit relevant sessile serrated lesions (SSLs) as neoplastic lesions 13 or use an endoscopy system that is not widely available. A study addressing all these issues would demonstrate realistic diagnostic performance of a CADx system when implemented in daily practice, which is an essential step before its widespread adoption in routine practice.

In the POLAR study, we aimed to address all these issues by developing a CADx system (POLAR system) that is able to characterize diminutive colorectal polyps, including SSLs, during real-time colonoscopy. The primary aim of the study was to compare the accuracy in optical diagnosis of diminutive colorectal polyps between the POLAR systems and a group of endoscopists during real-time colonoscopy. A secondary aim was to construct a publicly accessible colonoscopic imaging database that can be used to train, validate, or benchmark other CADx systems.

## Methods

### Setting and study design

This prospective, multicenter project was conducted from October 2018 to September 2021 in eight regional Dutch hospitals and one academic Spanish hospital in partnership with ZiuZ Visual Intelligence (Gorredijk, the Netherlands). The project comprised two phases: 1) development, (pre)training, and preclinical validation of the CADx system (POLyp Artificial Recognition [POLAR] system) for endoscopic characterization of diminutive colorectal polyps; 2) clinical validation of the system during live colonoscopies, compared with the performance of endoscopists participating in the Dutch or Barcelona Bowel Cancer Screening program (BCSP) 14 15 . The study is reported according to the Standards for Reporting of Diagnostic Accuracy Studies (STARD) statement 16 .

### Study outcomes

The primary outcome of the study was the comparison of accuracy in the optical diagnosis of diminutive colorectal polyps between the POLAR system and endoscopists (neoplastic [adenomas and SSLs] vs. non-neoplastic [hyperplastic polyps (HPPs)]) during the clinical validation (phase 2). Accuracy was defined as the percentage of correctly predicted optical diagnoses of the POLAR system or the endoscopists compared with the reference standard histopathology. For the calculation of the primary outcome, adenomas and SSLs were combined in the neoplastic category, and HPPs constituted the non-neoplastic category. There were multiple secondary outcomes in the study. 1) Sensitivity, specificity, negative predictive value (NPV), and positive predictive value [PPV] of the optical diagnosis of diminutive polyps were compared between the POLAR system and the endoscopists; different subgroup analyses were also performed (neoplastic vs. non-neoplastic and adenomas vs. SSL vs. HPPs). 2) Diagnostic accuracy was compared in a subgroup analysis for only high-confidence assessments. 3) The pooled NPV for neoplastic histology in the rectosigmoid and the surveillance interval agreement based on optical diagnosis of diminutive polyps with high confidence were compared between the POLAR system and endoscopists, respectively. Other secondary outcomes were the computation time of the POLAR system to diagnose a polyp, the number of images required for the POLAR system to diagnose a polyp, and the proportion of polyps in which POLAR was able to provide an optical diagnosis (i. e. success rate). Additional exploratory outcomes were: 1) the diagnostic accuracy of optical diagnosis of diminutive polyps if endoscopists were assisted by the POLAR system; 2) the evaluation of factors associated with accurate optical diagnosis of the POLAR system.

### Phase 1: development of the POLAR system



**Video 1** An example of our POLAR system. If the endoscopists detect a polyp, they take an image using narrow-band imaging. Subsequently, the system localizes the polyp, performs a quality check, and performs an optical diagnosis. In this video, the POLAR system predicts with high confidence that this is an adenoma (confidence level of 81 %).


**Video 2** An example of our POLAR system. If the endoscopists detect a polyp, they take an image using narrow-band imaging. Subsequently, the system localizes the polyp, performs a quality check, and performs an optical diagnosis. In this video the POLAR system predicts with high confidence that this is a hyperplastic polyp (confidence level of 91 %).


**Fig. 1:**
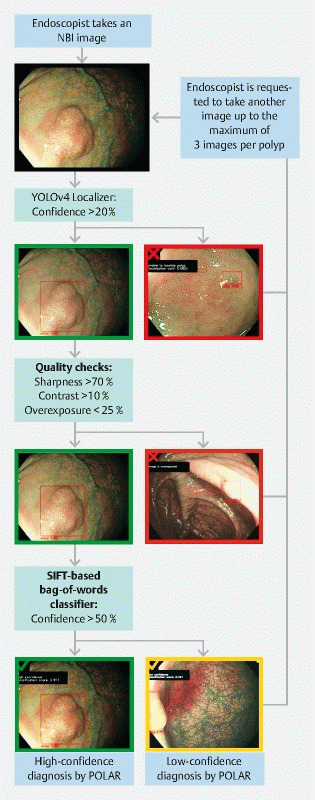
POLAR system design and user protocol during clinical validation. When using the POLAR system during clinical validation, the endoscopist has to take a maximum of three nonmagnified images using narrow-band imaging. After one image of the lesion is taken, it is processed by the POLAR system. If the system is able to provide a high-confidence diagnosis (a green mark is shown on the monitor), the endoscopist can continue with the procedure (i. e. resect the lesion). If the system is not able to provide a high-confidence diagnosis, the system provides feedback to the endoscopist on why this was not possible (e. g. not able to localize the lesion [red mark], not of sufficient quality [red mark], or only able to perform an optical diagnosis with low confidence [orange mark]). If the system is not able to provide a high-confidence diagnosis, the endoscopist has to take another image, up to a maximum of three per lesion. If the system is still not able to provide a high-confidence diagnosis after three images, the endoscopists can stop taking images, and proceed with the procedure. The low-confidence diagnosis with the highest prediction score is used as the final diagnosis of the system. If the system is not able to provide a low-confidence diagnosis after three images, this is considered as a failure of the system. NBI, narrow-band imaging.

**Fig. 2:**
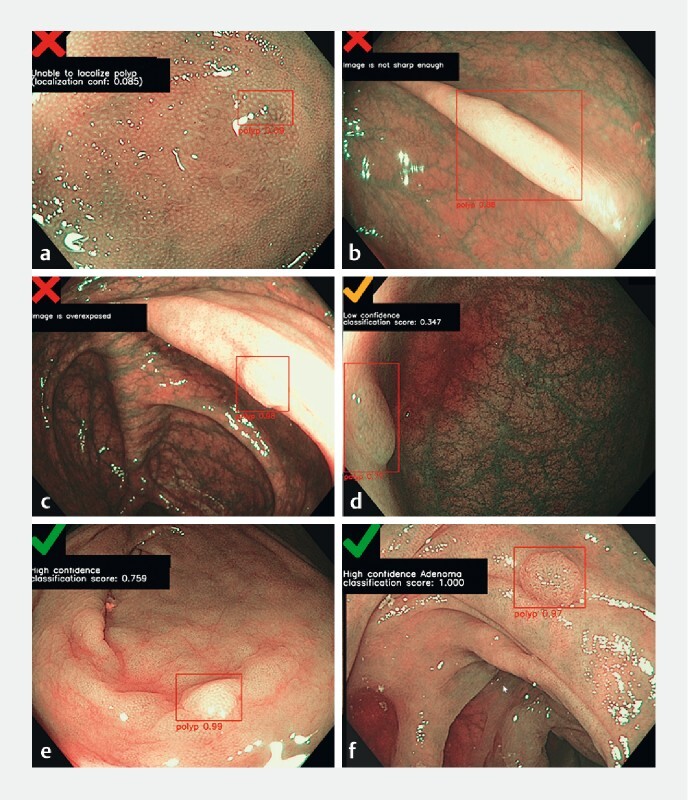
Examples of polyp images with the POLAR system output. **a** The POLAR system is not confident enough about the localization (8.5 %). **b, c** The area within the bounding box is not sharp enough or is overexposed. **d** The POLAR system predicts the histology of the polyp with only low confidence. **e, f** The POLAR system predicts the histology of the polyps with high confidence (blinded prediction [ **e** ], visible prediction [ **f** ]).

The POLAR system consists of three components: 1) a polyp localization model that outlines the polyp boundaries within each captured image (bounding box); 2) a quality parameter check that evaluates whether each captured image is of sufficient quality; 3) a polyp characterization model that differentiates between adenoma, SSL, and HPP ( Fig. 1 , Fig. 2 , Video 1 , Video 2 ).

#### Datasets

Models were initially pretrained on the MS COCO dataset that contains 330 000 images from a broad range of object classes 17 . Subsequently, the models were trained and preclinically validated with prospectively collected endoscopic images of polyps linked with histopathology from eight Dutch hospitals (see **Appendix 1 s** in the online-only Supplementary material) *.* This dataset consisted of 2637 narrow-band imaging (NBI) nonmagnified images, originating from 1339 unique histologically confirmed polyps (73 % adenomas, 10 % SSLs, and 17 % HPPs) found during 555 different colonoscopies.

#### Model development

For the localization stage, we used a convolutional neural network based on the YOLOv4 object detection model 18 . The classifier that consistently outperformed all other models in a fivefold cross validation setting was finally based on a Bag of Visual Words approach using SIFT features that are extracted from each sub-image by means of grid sampling (gridSIFT) 19 20 . Prediction scores by the classifier of ≥ 0.33 were deemed good enough for a low-confidence diagnosis. Prediction scores of ≥ 0.50 were deemed good enough for a high-confidence diagnosis 21 22 . **Appendix 2 s** provides an extensive description of the models used in the POLAR system.

### Phase 2: clinical validation of the POLAR system

#### Patients, endoscopists, and setting

Consecutive individuals undergoing screening colonoscopy following a positive fecal immunochemical test (FIT) and who provided informed consent were eligible to participate in the clinical validation of the POLAR system. Exclusion criteria included inflammatory bowel disease, Lynch syndrome, and polyposis syndromes. Endoscopists were required to be accredited for performing colonoscopies within the BCSP. (During the accreditation process, practical skills are evaluated, and knowledge and achievement of evidence-based quality indicators are measured.) At each center, up to three endoscopists were invited to participate in the clinical validation phase; all were required to perform at least 10 study procedures. All procedures had to be performed with EVIS EXERA II or III video processors with 190-series Olympus colonoscopes containing NBI (Olympus, Tokyo, Japan).

#### Endoscopy procedure and data collection

All procedures were performed according to the local hospital protocol. For each polyp, location, size, and morphology (Paris classification 23 ) and predicted histology were recorded by the endoscopist. Histology of the detected lesions was predicted including a high- or low-confidence assessment (HPP, SSL, adenoma, carcinoma, or other). After scoring, endoscopists had to follow the “POLAR system user protocol” ( Fig. 1 , Fig. 2 , Video 1 , Video 2 ). This user protocol was designed to develop a system that is easy to use in daily practice. In addition, this user protocol ensured that during the validation phase, all endoscopists used the system in the same way. During the study procedure, the histology prediction of POLAR was not visible to the endoscopists. Endoscopists were instructed to remove all lesions, except for multiple (≥ 3) diminutive HPPs in the rectosigmoid. The latter could be left in situ but the endoscopist was instructed to biopsy or remove at least one polyp representing the sample. Procedural findings were codified and registered by the study coordinators in a secure online database (www.castoredc.nl; Castor Electronic Data Capture, Amsterdam, the Netherlands).

#### Histopathology

All lesions were assessed by pathologists with expertise in gastrointestinal pathology in the local hospital. Histopathological assessment was performed according to the 2010 World Health Organization classification 24 . The Dutch pathologists were all accredited for the national BCSP. This accreditation process included e-learning on characterization of serrated polyps 25 .

#### Hypothesis and sample size calculation

The required sample size was calculated by using a two-sided McNemar test with 90 % power and 0.05 alpha, with the null hypothesis that the two marginal probabilities for each outcome in the 2 × 2 contingency table are the same (i. e. the performance of the POLAR system and the endoscopist are similar).

The clinical validation study was designed to find a 10 % difference in diagnostic accuracy of optical diagnosis between the POLAR system and the participating screening endoscopists. Based on previous research, we assumed a difference between the optical diagnosis by the POLAR system and the endoscopists in 20 % of 1–5 mm polyps (regardless of whether this optical diagnosis was correct or not) 26 . Using these parameters, at least 206 diminutive polyps were required to detect a statistical difference in the accuracy. Assuming that a mean of 0.74 (SD 1.23) diminutive polyps would be detected during each colonoscopy 22 , and allowing a patient dropout rate of 5 %, the projected sample size was 292 patients. The sample size was calculated using R, package Trialsize (R Foundation for Statistical Computing, Vienna, Austria. www.R-project.org ).

#### Statistical analysis

The difference in pooled accuracy, sensitivity, and specificity of optical diagnosis between the POLAR system and endoscopists was compared using two-sided McNemar tests with continuity correction. The PPV and NPV were compared using the weighted generalized score and Fisher’s exact test. If the histopathology outcome was carcinoma, traditional serrated adenoma, normal mucosa, inflammatory lesion, or missing, the lesion was excluded from the diagnostic accuracy analysis. For all outcomes on diagnostic test accuracies, 95 %CIs were calculated. The statistical analysis for the other analyses can be found in **Appendix 3 s** . Analyses was performed in statistical software R (version 4.0.3). *P* values of less than 0.05 were considered statistically significant.

### Ethical approval, patient and public involvement, and role of the funding source

The Institutional Review Board of the Amsterdam University Medical Centre, location AMC, decided that formal revision was not required according to the Medical Research Involving Human Subjects Act (WMO) because patient data were retrieved during standard care without any interventions (10–01–2019, W18_422). Patients, the public, and sponsors were not involved in the design, conduct, reporting, or dissemination plans of our research. All contributing authors had access to the study data, and reviewed and approved the final manuscript.

### Construction of the publicly available POLAR database

The colorectal polyp image data collected during the training and validation of the POLAR system were used to construct a public database ( www.polar.amsterdamumc.org ). This database can be used for the development, validation, and benchmarking of other noncommercial AI systems.

## Results

### Baseline characteristics

**Fig. 3:**
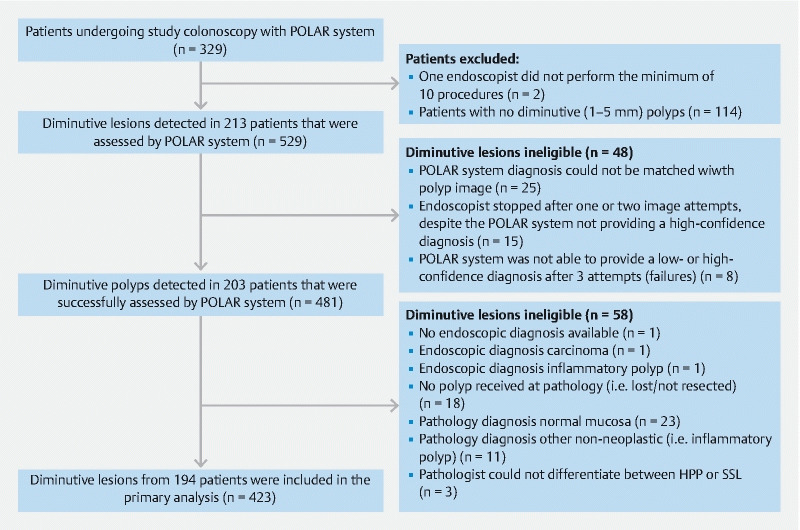
Study flow chart. CADx, computer-aided diagnosis; SSL, sessile serrated lesion; HPP, hyperplastic polyp.

Between April and September 2021, the diagnostic accuracy of the POLAR system was clinically validated and compared with the diagnostic accuracy of 20 screening endoscopists from eight hospitals ( Fig. 3 ). A total of 423 diminutive polyps from 194 FIT-positive patients were included for analysis ( **Table 1 s** , **Table 2 s** ). The median number of included colonoscopies per endoscopist was 10 (interquartile range [IQR] 7–10) and the median number of diminutive polyps per endoscopist was 21 (IQR 16–27).

### Study outcomes

#### Accuracy for optical diagnosis of diminutive polyps (neoplastic vs. non-neoplastic)

**Fig. 4:**
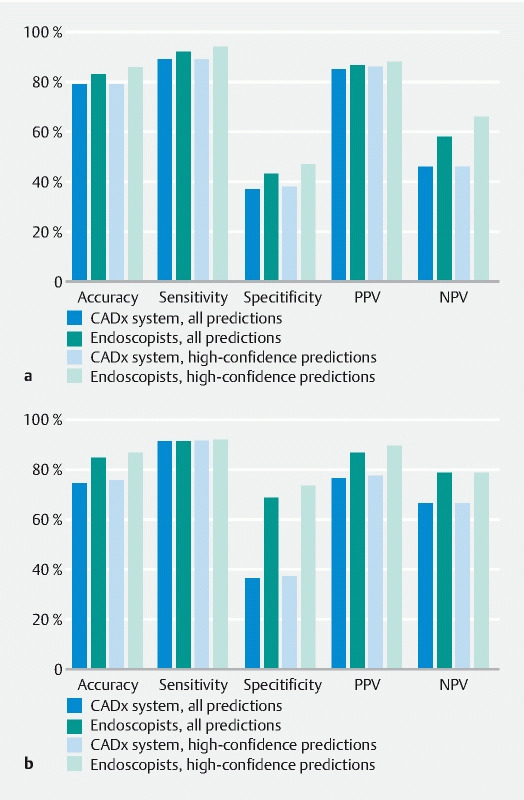
Diagnostic performance of both the computer-aided diagnosis system and endoscopists for differentiating neoplastic from non-neoplastic diminutive lesions. **a** Diminutive lesions overall. **b** Diminutive lesions located in the rectosigmoid. CADx, computer-aided diagnosis; PPV, positive predictive value; NPV, negative predictive value.

The POLAR system correctly diagnosed 305 of the 341 neoplastic diminutive lesions and 31 of the 82 non-neoplastic diminutive lesions ( Table 1 , Fig. 4 ). The accuracy, sensitivity, and specificity for optical diagnosis of the POLAR system were 79.4 % (95 %CI 75.2 %–83.2 %), 89.4 % (95 %CI 86.2 %–92.7 %), and 37.8 % (95 %CI 27.3 %–48.3 %), respectively. The endoscopists correctly diagnosed 315 of the 341 neoplastic diminutive polyps and 36 of the 82 non-neoplastic diminutive polyps. The accuracy, sensitivity, and specificity for the optical diagnosis of the endoscopists overall were 83.0 % (95 %CI 79.1 %–86.4 %), 92.4 % (95 %CI 89.6 %–95.2 %), and 43.9 % (95 %CI 33.2 %–54.6 %), respectively. The optical diagnosis accuracy between the POLAR system (79.4 %; 95 %CI 75.2 %–83.2 %) and the endoscopists (83.0 %; 95 %CI 79.1 %–86.4 %) was not significantly different ( *P* = 0.10). The overall accuracy for high-confidence predictions of neoplastic lesions by POLAR was 79.6 % (95 %CI 75.5 %–83.3 %) compared with 85.8 % (95 %CI 81.8 %–89.2 %) by endoscopists.

**Table 1:** Diagnostic performance of both the computer-aided diagnosis system and endoscopists for differentiating neoplastic from non-neoplastic diminutive lesions. 1

	Overall		Proximal to rectosigmoid		Rectosigmoid	
	CADx	Endoscopists	CADx	Endoscopists	CADx	Endoscopists
All predictions, n	423	423	301	301	122	122
All predictions, % (95 %CI)						
Accuracy	79.4 (75.2–83.2)	83.0 (79.1–86.4)	81.4 (76.5–85.6)	82.4 (77.6–86.5)	74.6 (65.9–82.0)	84.4 (76.8–90.4)
Sensitivity	89.4 (86.2–92.7)	92.4 (89.6–95.2)	88.7 (84.9–92.3)	92.6 (89.4–95.8)	91.4 (85.4–97.5)	91.7 (85.8–97.6)
Specificity	37.8 (27.3–48.3)	43.9 (33.2–54.6)	38.6 (24.3–53.0)	22.7 (10.3–35.1)	38.8 (22.9–54.8)	68.4 (53.6–83.2)
PPV	85.7 (82.0–89.3)	87.3 (83.8–90.7)	89.4 (85.6–93.2)	87.5 (83.6–91.4)	77.3 (69.0–85.7)	86.5 (79.4–93.6)
NPV	46.3 (34.3–58.2)	58.1 (45.8–70.4)	34.5 (23.0–50.9)	34.5 (17.2–51.8)	66.7 (46.5–86.8)	78.8 (64.8–92.7)
Only high-confidence predictions, n	422	367	301	263	121	101
Only high-confidence predictions, % (95 %CI)						
Accuracy	79.6 (75.5–83.3)	85.8 (81.8–89.2)	81.4 (76.5–85.6)	85.6 (80.7–89.6)	75.2 (66.5–82.6)	86.9 (79.0–93.7)
Sensitivity	89.4 (86.2–92.7)	94.7 (92.1–97.2)	88.7 (84.9–92.6)	95.6 (92.9–98.3)	91.7 (85.8–97.6)	91.9 (85.7–98.1)
Specificity	38.3 (27.7–48.9)	47.1 (35.2–58.9)	38.6 (24.3–53.0)	26.3 (12.3–40.3)	37.8 (22.2–53.5)	73.3 (57.5–89.2)
PPV	85.9 (82.3–89.5)	88.7 (85.2–92.2)	89.4 (85.6–93.2)	88.5 (84.4–92.5)	77.0 (68.7–85.3)	89.5 (82.6–96.4)
NPV	46.3 (34.3–58.2)	66.7 (53.3–80.0)	37.0 (23.0–50.9)	50.0 (28.1–71.9)	66.7 (46.5–86.8)	78.6 (63.4–93.8)

CADx, computer-aided diagnosis; PPV, positive predictive value; NPV, negative predictive value.

1 For the calculation of the diagnostic accuracies (neoplastic vs. non-neoplastic), adenomas and sessile serrated lesions (SSL) were considered neoplastic polyps, while hyperplastic polyps were considered non-neoplastic. Note that when an SSL was assessed as an adenoma or vice versa, this was also considered

#### Other diagnostic test accuracies for optical diagnosis

Table 2 shows that the accuracy to differentiate between adenomas, SSLs, and HPPs (i. e. per polyp subtype accuracy) by POLAR was 73.1 % (95 %CI 68.6 %–72.2 %) compared with 76.1 % (95 %CI 71.8 %–80.1 %) by endoscopists. For SSLs vs. no SSLs, optical diagnosis assigned with high confidence resulted in a sensitivity of 17.1 % (95 %CI 5.6 %–28.6 %) by POLAR and 58.5.6 % (95 %CI 43.5 %–73.6 %) by endoscopists. The accuracy of CADx-assisted optical diagnosis for high-confidence assessments only was 84.1 % (95 %CI 80.2 %–87.5 %), while the sensitivity and specificity were 93.2 % (95 %CI 90.5 %–95.9 %) and 44.9 % (95 %CI 33.8 %–55.9 %), respectively ( **Table 3 s** ).

**Table 2:** Diagnostic performance of the computer-aided diagnosis system and endoscopists in differentiating diminutive colorectal polyps.

	Overall		Only high-confidence predictions	
	CADx (n = 423)	Endoscopists (n = 423)	CADx (n = 422)	Endoscopists (n = 367)
Per polyp subtype accuracy 1	73.1 (68.6–77.2)	76.1 (71.8–80.1)	73.2 (68.7–77.4)	79.8 (75.4–83.8)
Adenoma vs. nonadenoma (i. e. serrated), % (95 %CI)				
Accuracy	77.8 (73.3–81.7)	82.7 (78.8–86.2)	78.0 (73.7–81.8)	85.0 (80.9–88.5)
Sensitivity	90.3 (87.0–93.7)	87.3 (83.6–91.1)	90.3 (87.0–93.7)	89.9 (86.2–93.5)
Specificity	47.2 (38.3–56.0)	71.5 (63.6–79.5)	47.5 (38.7–56.4)	72.3 (63.6–81.0)
PPV	80.6 (76.4–84.9)	88.2 (84.6–91.9)	80.9 (76.7–85.1)	89.5 (85.8–93.2)
NPV	66.7 (56.8–76.6)	69.8 (61.8–77.9)	66.7 (56.8–76.6)	73.0 (64.3–81.7)
SSL vs. non-SSL, % (95 %CI)				
Accuracy	88.9 (85.5–91.7)	88.8 (85.2–91.9)	88.9 (85.5–91.7)	88.8 (85.2–91.9)
Sensitivity	17.1 (5.6–28.6)	58.5 (43.5–73.6)	17.1 (5.6–28.6)	67.7 (50.6–82.8)
Specificity	96.6 (94.8–98.4)	89.5 (86.5–92.6)	96.6 (94.8–98.4)	91.0 (88.0–94.1)
PPV	35.0 (14.1–55.9)	37.5 (25.6–49.4)	35.0 (14.1–55.9)	42.3 (28.9–55.7)
NPV	91.6 (88.9–94.3)	95.3 (93.0–97.5)	91.5 (88.8–94.3)	96.5 (94.5–98.5)

CADx, computer-aided diagnosis; PPV, positive predictive value; NPV, negative predictive value; SSL, sessile serrated lesion.

1 For the calculation of the per polyp subtype accuracy adenomas, SSLs and hyperplastic polyps were considered as different histological subtypes.

#### NPV of neoplastic lesions in the rectosigmoid and surveillance interval agreement (PIVI)

The NPV for optically diagnosing diminutive neoplastic polyps in the rectosigmoid with high confidence was 66.7 % (95 %CI 46.5 %–86.8 %) by POLAR and 78.6 % (95 %CI 63.4 %–93.8 %) by endoscopists ( Table 1 ). The pooled surveillance interval agreement was 96.1 % (95 %CI 81.8 %–98.6 %) in the endoscopist group and 95.5 % (95 %CI 90.9 %–98.2 %) with POLAR.

#### Success rate and computation time of the POLAR system

The proportion of all polyps in which POLAR was able to provide an optical diagnosis within the maximum of three images was 98.3 % (95 %CI 96.7 %–99.2 %; 481 /489 diminutive polyps). The system required only one image to diagnose the lesion with high confidence in 95 % of polyps, two images in 4 % of the polyps, and three images in 1 % of the polyps. The mean duration for an optical diagnosis per image was 1.4 seconds (SD 0.6).

#### Factors associated with accurate optical diagnosis by the POLAR system

Several factors where independently associated with accurate histology prediction by the POLAR system in a multivariate analysis: a polyp size of 1–2 mm, a polypoid morphology, a classifier confidence score of ≥ 0.8, and the 10 endoscopists with the highest optical diagnosis accuracy ( **Table 4 s** ).

## Discussion

To improve the accuracy and reliability of endoscopists’ optical diagnosis of diminutive (1–5 mm) colorectal polyps, numerous CADx systems based on machine learning techniques have been developed 7 8 9 10 11 . Although several systems show impressive results for optical diagnosis in prospective settings, work is still required before being implemented and accepted in daily practice. Such a CADx system should first be validated during live endoscopy in a prospective and multicenter setting, include the SSLs, which are now recognized as being important lesions, and compared with a group of representative endoscopists 27 . Results of such a study would demonstrate its real diagnostic performance to the endoscopy community when implemented in daily practice. In this article, we therefore present the rigorous development, clinical validation, and benchmarking of a CADx system, POLAR (POLyp Artificial Recognition). The system performs real-time optical diagnosis of diminutive colorectal polyps using a maximum of three NBI images taken during colonoscopy, and includes optical diagnosis of SSLs. The validation setting, with broad and diverse data, and with a comparison with endoscopists from multiple hospitals, provides robust data and demonstrates the reliability of our findings for daily practice. In this study, we also constructed the publicly accessible POLAR database that can be used by other researchers for the development and validation of their CAD systems.

During real-time endoscopy, the POLAR system achieved an accuracy of 79 % for differentiating neoplastic from non-neoplastic diminutive polyps, an accuracy that was comparable to that of 20 screening endoscopists from eight hospitals. Neither the system nor the endoscopists were able to achieve the SODA-1, SODA-2, or PIVI-2 competence standards required for safe implementation of the optical diagnosis strategy for diminutive polyps 3 4 . POLAR did not meet these standards due to a suboptimal specificity of 38 % and an NPV in the rectosigmoid of 67 % for diagnosis of diminutive neoplasia with high confidence. Given the strict methodology for validation of the POLAR system, and the fact that two recently published multicenter trials also showed that CADx systems alone were not able to meet all competence thresholds, our results were not unexpected 9 10 . If we aim to introduce such a system in the future, it is relevant to understand the underlying reasons for the suboptimal specificity and NPV (when using such strict study methodology). We could then aim to improve the performance of the current POLAR system, and potentially also other systems.

Apart from the strict validation approach, another explanation for the suboptimal specificity and NPV may be the fact that our study is one of the few to also include the optical diagnosis of SSLs in the CADx system 28 29 . Including SSLs is of importance because, like adenomas, SSLs can also develop into cancer via the serrated neoplasia pathway 30 . As such, differentiating SSLs from adenomas and HPPs is of importance for the implementation of the optical diagnosis strategy. POLAR was able to differentiate between adenomas, SSLs, and HPPs with an accuracy of 73 %, which was comparable to the accuracy of 76 % achieved by endoscopists. For SSLs, optical diagnosis by POLAR resulted in an unsatisfactory sensitivity of 17.1 %. Excluding diminutive SSLs from the diagnostic accuracy analysis resulted in only a slight improvement for optical diagnosis by POLAR ( **Table 5 s** ) *.* To explore whether the known pathological interobserver variability during histopathological evaluation may have played a role in the suboptimal accuracy (especially between SSL and HPP) 31 , we also evaluated the diagnostic performance of POLAR on the best “gold standard” pathology (adenoma vs. serrated). However, in this subanalysis the performance of POLAR barely improved (adenoma vs. serrated; specificity from 38 % to 48 % and NPV rectosigmoid remained at 67 %).

We believe we have reached the point at which big improvements in the performance of our model (and potentially also other systems) are nearly impossible with the data that we currently have. The performance of the models could be improved by increasing the quantity of data, but more importantly by improving the variety and quality of the data. Even though the dataset that was used to train and test the system in this study was broad and diverse, we noticed that some polyps were more difficult to collect in daily practice because they are relatively rare or not removed in clinical practice (e. g. diminutive HPPs in the rectosigmoid). These rare or not removed polyps might be even more relevant for the development of a CADx system. Although we have done our utmost to collect high quality training data in a standardized manner, we do believe that even more rigorous data collection should be done. This hypothesis was supported by our multivariate regression analysis, which demonstrated that POLAR performed better when endoscopists with the highest accuracy for optical diagnosis were taking the images. This suggests that there are differences between the quality of the images collected by the best and least performing endoscopists. We strongly believe that if these differences can be identified, they would yield extremely useful insights as to how we can improve the quality of image collection and build better performing CADx systems.

Potential limitations of this study should also be mentioned. First, our CADx system was specifically trained and validated only on NBI. As white-light endoscopy is the most basic and available endoscopy diagnostic modality, application of CADx to white-light endoscopy would be beneficial 32 33 . A second limitation is that our CADx system was image based, whereas video-based CADx systems may be considered more state of the art. However, disadvantages of a video-based, frame-by-frame CADx system are the processing time, interlacing, and motion blur. Another potential limitation is that no intracluster correlation (e. g. of 0.05) was included in the sample size calculation, thereby not correcting for the fact that some polyps were detected in the same patient and thus may introduce statistical dependencies. Finally, we chose not to perform a central histopathology review. Histopathology reading of diminutive polyps is associated with variable levels of interobserver agreement. In our study, every Dutch pathologist performing histological analysis for the national FIT-based screening program was obliged to complete and pass an obligatory e-learning module. The Spanish pathologist was a highly devoted expert in gastrointestinal pathology. Therefore, we believe the quality of pathological analysis in this study was high.

To conclude, we report the development and validation of a CADx system, suitable for performing optical diagnosis of diminutive colorectal polyps during endoscopy, using a maximum of three NBI images. In a multicenter setting, the system achieved an accuracy for neoplastic diminutive polyp diagnosis that was comparable to that of a group of screening endoscopists. The methodology of this study provides a reliable diagnostic accuracy of the POLAR system in daily practice. To increase the diagnostic accuracy of this and other CADx systems, efforts should be made to improve the variety, but most importantly the quality, of data.

## Supplementary material

Supplementary material Zusatzmaterial
