# Supplementary material for: Computer-aided diagnosis for optical diagnosis of diminutive colorectal polyps including sessile serrated lesions: a real-time comparison with screening endoscopists
Source: Endoscopy. 2023 Mar 2;55(8):756–65. doi: 10.1055/a-2009-3990 (PMC10374350; doi:10.1055/a-2009-3990)
Supplement: Supplementary file 3 — Supplementary material [file 22262supmat_10-1055-a-2009-3990.pdf]

Supplementary material

Computer-aided diagnosis for optical diagnosis of diminutive colorectal polyps including sessile serrated lesions: a real-time comparison with screening endoscopists

B.B.S.L. Houwen, Y. Hazewinkel, I. Giotis, J.L.A. Vleugels, Nahid S. Mostafavi, P. van Putten, P. Fockens and E. Dekker on behalf of the POLAR Study Group

Supplementary material

Table of contents

Appendix 1s Standardized image acquisition protocol prospective data collection for training and internal validation 3

Appendix 2s Development CAD system models 6

Appendix 3s Statistical analysis for the negative predictive value (NPV) of neoplastic lesions in the rectosigmoid and agreement of the assigned surveillance intervals 13

Table 1s Patient characteristics and colonoscopy quality characteristics 15

Table 2s Characteristics of the diminutive lesions in the clinical validation set 15

Table 3s Diagnostic performance of both CADX system, endoscopists and CADx-assisted for differentiating neoplastic from non-neoplastic diminutive lesions[a] 16

Table 4s Outcomes of multilevel logistic regression analysis to find factors associated with accurate optical diagnosis by POLAR 17

Table 5s Diagnostic performance of CADx system and endoscopists in differentiating diminutive colorectal polyps (adenomas vs hyperplastic polyps)[a] 18

Supplementary material

Appendix 1s Standardized image acquisition protocol prospective data collection for training and internal validation

A. Colonoscopy procedure

I. The endoscopist or nurse checks whether the patient signed informed consent.
II. The endoscopist or nurse starts the study tablet (POLAR tablet) by selecting "start treatment". The tablet will automatically generate a study number (**Figure 1.1s**).
III. The endoscopist or nurse notes on a separate confidential linking list the study number and patient characteristics (name, date of birth and sex).
IV. The endoscopist or nurse selects the type of colonoscope and type of video processor used on the tablet.
V. If a polyp is detected, the endoscopist has to take at least two images of each polyp from the front with NBI without magnification. These endoscopy images will be transferred automatically to the storage box and study tablet (POLAR box and tablet). The storage box shows the last endoscopy images on the study tablet.
VI. The endoscopist or nurse selects the correct photos of the polyp on the tablet. Only these photos will be stored on the storage box (Figure **1.2s**).
VII. The endoscopist or nurse also records on the tablet the number of the pathology container in which the lesion is collected. The storage system stores this combination of image, type of colonoscope, type of video processor, pathology container number and study number.
VIII. Subsequently, the polyp can be removed at the discretion of the endoscopist.
IX. The endoscopy nurse collects each lesion in a separate pathology container, and sends it to the pathologist for histopathological examination.
X. The endoscopist or nurse finishes the session by selecting "end of treatment" on the tablet.

B. Procedure to collect pathology results of participants:

After patients are successfully enrolled and images of polyps have been collected, the corresponding pathology results from these patients are collected through the national PALGA database (Pathologisch-Anatomisch Landelijk Geautomatiseerd Archief, https://www.palga.nl/). **Figure 1.3s** shows an overview of the anonymization process.

I. Every participating center receives software from ZorgTTP (https://www.zorgttp.nl/) to pseudonymise the list of included study patients (name, date of birth and sex), including an administration number.
II. These pseudonymised data will be sent to ZorgTTP through a secure link. ZorgTTP is a Thrusted Third party, which provides the pseudonymization for PALGA.
III. ZorgTTP performs a second pseudonymization and send this pseudonymised data including administration number to PALGA.
IV. PALGA links the pseudonyms of the research cohort to the pseudonyms in the PALGA database and selects the requested pathology results.
V. PALGA links the pathology results with these pseudonymised data and sends it back to the participating center.

C. Procedure connecting pathology-endoscopic image:

I. The local study investigator links the pathology results list from PALGA (pathology and patient numbers) with the linking list (study number, patient number). The local study

Supplementary material

investigator deletes the patient number after combining these two lists. This pathology-study number list will be stored on the secured POLAR laptop.

II. Secondly, the local study investigator exports the endoscopic images and study numbers from the POLAR box to the secured POLAR laptop.

III. After, the local study investigator merges the pathology-study number list with the endoscopic images-study numbers.

This procedure is performed every 1-2 months at the local study site by the local study investigator (supplier) on a secured laptop in collaboration with the Amsterdam UMC.

D. Anonymization procedure:

I. Finally, when the endoscopic images and pathology results are merged, the data will be anonymized with the use of an anonymization tool. This anonymization tool removes study numbers, metadata and changes a random pixel on the corner of the image to a random other pixel. The anonymization tool runs on the secured laptop by the local study investigator in collaboration with the Amsterdam UMC. After, the linking list will be removed by the local investigator.

II. The anonymization procedure will be double-checked by the local study investigator (supplier) and recipients.

III. After checking the anonymization procedure of the data, the anonymized set of images will be delivered on a secured hard drive together with polyp pathology type, type of colonoscope and video processor. These anonymized data re transferred to the Amsterdam UMC and ZiuZ.

After all images are collected in the storage system, the CAD system will be further trained and tested with these additional data.

Fig. 1.1s

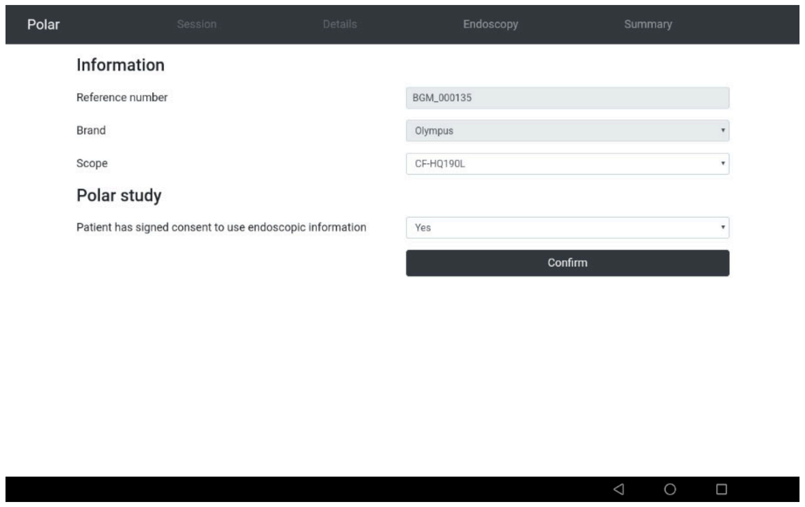

Thieme

Supplementary material

Fig. 1.2s

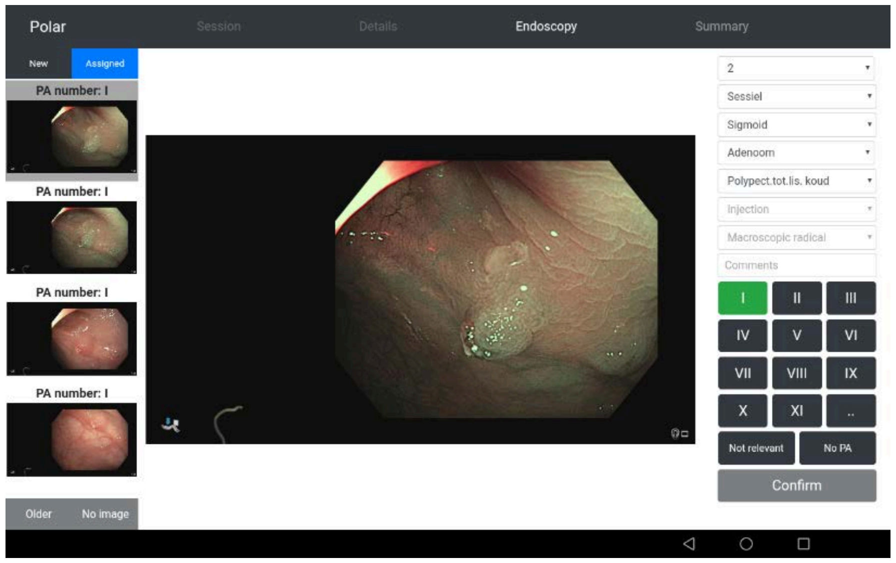

Fig. 1.3s

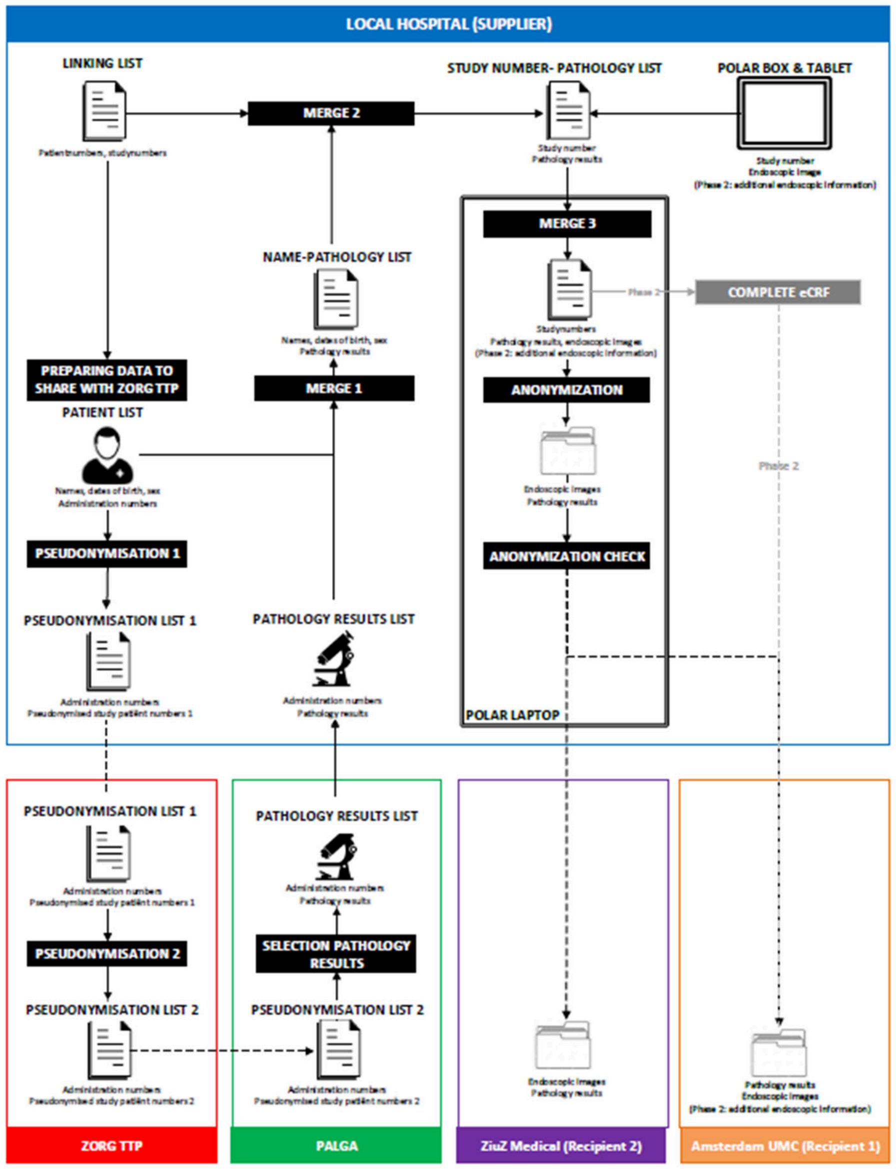

Supplementary material

Appendix 2s Development CAD system models

The CAD system we developed consists of three main components: (1) polyp localization, i.e. outlining the polyp boundaries within each image, (2) quality checks, and (3) polyp classification.

Polyp localization

To localize a polyp within an endoscopic image, a convolutional neural network based on the YOLOv4 object detection model was trained.[1] Object detection models are trained to look at an image and search for a subset of object classes. When found, these object classes are enclosed in a bounding box and their class is identified. In our case there was only one class of objects we aimed to identify; that was the class polyp and therefore we have tweaked the YOLOv4 model to always return the bounding box that was most likely to contain a polyp.

YOLOv4 is a single shot object detection model. Such models are rather complex neural networks, but they typically consist of three main stages: the backbone, the neck, and the head. The backbone is in most cases a pre-trained, convolutional neural network (CNN) that is able to learn features from raw images and yield a class prediction. The CNN used as a backbone in YOLOv4 is CSPDarknet53. It is based on Darknet53[2], a CNN with successive 3x3 and 1x1 convolutional layers as well as a multitude of skip connections among non-successive layers (DenseNet) in order to mitigate the problem of vanishing gradients and reduce the number of trainable network parameters.[3] Compared to Darknet53, the CSPDarknet53 variant employs cross stage partial connections that integrate the gradient changes into the feature maps across the whole network, thus reducing the number of calculations needed while ensuring accuracy.[4] The neck part of an object detector is used to collect and aggregate feature maps from different stages of the backbone and is composed of several bottom-up and several top-down paths. YOLOv4 chooses the Path Aggregation Net (PANet)[5] for feature aggregation and adds a spatial pyramid pooling (SPP) [6] block after the CSPDarknet53 backbone to increase its receptive field. The pyramid topology is especially powerful because it allows YOLOv4 to detect objects of different sizes. Blocks with smaller spatial resolution are able to detect large objects, whereas blocks with larger resolution specialize in detecting small objects. Since, the features from different resolutions are concatenated, the fully aggregated features at the end of the feature extractor are capable to detect objects of different scales and sizes. Finally, the head of the object detector is the part responsible for making the predictions; that is assigning bounding boxes and a set of conditional object class probabilities at specific coordinates on the image. This is done by first splitting the image in a grid of cells and subsequently predicting a fixed number of bounding boxes within each cell as well as the corresponding conditional class probabilities and a confidence score that represents the overlap between the predicted bounding box and any ground truth box. Combining the bounding boxes and the confidence scores thereof with the class probabilities yields the final predictions for object detection as shown in **Fig 2.1s**.[7]

The YOLOv4 model we used for our CAD system was initially trained on the MS COCO dataset that contains more than 300k images from a broad range of object classes (80 in total), such as person, window, car, and chair.[8] Since YOLOv4 is able to learn features that generalize very well, we initially used the pre-trained backbone and neck parts of the neural network as is and we only trained the head part for the object classes we were interested in (domain adaptation). We initialized the weights of the head part of the network with the final weights of its pre-trained version and we trained further for a number of epochs (or until convergence is reached). In our case, we were only interested in one

Supplementary material

object type; that was polyps. At this point the YOLOv4 model we have trained was able to detect polyps, but we opted for one more, final training phase. In this training phase we further trained all neural network layers, only this time with a very small learning rate in order to fine-tune the model. This procedure of changing the domain of pre-trained neural network and then fine tuning to further improve its performance is called transfer learning.[9] The output of the localizer finally consisted of the bounding box of the area most likely to contain a polyp and a score between 0 and 1, representing the likelihood that a polyp lies within this bounding box. For the final CAD system, scores of ≥0.2 were deemed to be good enough to accept a localization. This threshold has been empirically chosen based on the cumulative distribution function of the localization confidence scores in dataset 2 (**Figure 2.2s**), which we obtained using 5-fold cross validation. Setting the threshold at 0.2 led to an Intersection over Union (IoU) score of 80.7%, a Dice coefficient of 87.8%, and an acceptable data loss, namely the loss of roughly 5.7%. Each polyp in the training datasets was annotated with a bounding box by two non-medical experts, who consulted a medical expert in difficult cases.

<u>Quality checks</u>

In case of a successful localization, the area defined by the bounding box (sub-images) was evaluated with regard to three parameters: sharpness, contrast and percentage of overexposed pixels. This was done to ensure that only images of sufficient quality are presented to the classification stage of the pipeline. We defined sharpness as the variance of the Laplacian of the image.[10] The Laplacian operator highlights regions of an image containing rapid intensity changes. We set the threshold for sufficient sharpness to 70 after empirically evaluating a number of blurry images in our dataset and their corresponding sharpness values. Contrast was defined as the standard deviation of the intensity values of an image (Root mean scare contrast). Contrast is directly related to brightness and images with insufficient contrast are perceived as too dark from the human visual system. We set the minimum acceptable contrast equal to 10 in the same empirical manner as before. Finally, we defined as overexposed all those pixels with an intensity value >245 (the upper 4% of the dynamic range) across all three color channels. If a sub-image contained more than 25% overexposed pixels it was considered of insufficient quality. These thresholds were also set by visually evaluating random images from our dataset. Setting the quality check thresholds at these values resulted to the loss of roughly another 6% of the images. Consequently, our processing pipeline was able to reach the classification stage for approximately 88% of the input training images.

<u>Polyp classification model</u>

In case of a successful localization and sufficient quality, the area defined by the bounding box (sub-image) was evaluated by the classifier. The aim of the classifier in the POLAR system was to automatically differentiate adenomas, SSLs and hyperplastic polyps, similarly to the WASP classification for optical diagnosis.[11] In our search for the model with best performance, we compared a number of different classifiers including local image descriptors such as scale-invariant feature transform (SIFT) or Local Binary Pattern[12], as well as popular CNN architectures such as the VGG, AlexNet and DenseNet.[3, 13, 14] The classifier that consistently outperformed all other classification models in the aforementioned 5-fold cross validation setting is finally based on the local features approach proposed by Tamaki et al[15], which we have adapted and parameterized according to our dataset and classification problem (**Figure 2.3s**).

Supplementary material

The core of our classifier relies on SIFT descriptors[16] extracted from each image using grid sampling (gridSIFT).[17] SIFT is a local feature descriptor invariant to shift, rotation, scale, and intensity changes that essentially encodes the image gradients of various orientations within a neighborhood of pixels. In the traditional approach sparse points of interest are detected on an image and the SIFT descriptors are extracted only at those points, whereas in our approach the image was split in a regular grid and SIFT descriptors for multiple scales and orientations were extracted from each cell of the grid (**Figure 2.4s**).[18]

Our gridSIFT implementation contained three pre-processing steps. First, we defined our grid to ignore 15% of the pixels at the periphery of the images, based on the empirical observation that the bounding boxes from the localization step tend to position the polyps in their center, but included always background pixels. Subsequently, we applied a local contrast enhancement technique called unsharp masking that allowed us to amplify the high frequency components of the image. Unsharp masking consists of computing a gaussian blurred version of the original image and then creating a new, sharpened image using the blending formula ***sharpened = original + (original − blurred) × amount***. We obtained the best results when using a Gaussian filter with $\sigma=2$ and we set ***amount=0.5***. Finally, we designated all pixels with values in the upper 4% of the dynamic range in all color channels as overexposed and excluded them from the gridSIFT feature extraction.

We used an initial grid spacing of 11 pixels for images up to 525x525 pixels. This grid spacing was enlarged by 11 pixels every time the image size became at least 66% larger with respect to one (or more) of its dimensions. Since smaller spacing means more features, we ensured that larger images would not generate a bigger number of features to such a degree that they would introduce a bias in our training data. **Figure 2.5s** depicts the locations of the key points within a grid that was automatically created for a random image in our dataset. At every grid point we specified a range of scales (5, 7, 9 and 11 pixels) and orientations (0, 45, 90, 135 and 180 degrees) to extract features from. **Figure 2.6s** shows an example of the spatial information that key points on the same grid point but with different scales will take into account. From every grid point we extracted 20 SIFT descriptors (one for every combination of scale and orientation), which added up to ***N*20*** feature descriptors for an image with ***N*** grid cells. In our dataset (dataset 2) we had on average **320 grid points** and thus **6400 SIFT descriptors per image** ($4 \leq N \leq 1026$).

In the next step we used the extracted SIFT descriptors to train a Bag-of-Visual-Words (BoVW) representation of the polyp images. We chose the total size of our vocabulary to be 4500 visual code words, and explicitly assigned a specific amount of code words to represent each class of polyps according to their distribution across the examples (17% hyperplastic polyp, 10% sessile serrated lesion and 73% adenoma). This yielded 465 code words for hyperplastic polyps, 450 code words for sessile serrated lesions and 3285 code words for adenomas. To create these code words we used the mini-batch k-Means clustering algorithm.[19] The SIFT descriptors from the images of each class of polyps were clustered separately with k = 465, 450 and 3285 for hyperplastic polyp, sessile serrated lesion and adenoma, respectively. The resulting cluster centroids are vectors in the same feature space as the SIFT descriptors and served as the code words of our visual vocabulary. Finally, we mapped every original SIFT descriptor to a certain code word through the clustering process, thus creating a histogram of code words for every image in the dataset. Ultimately, we used the normalized histogram

Supplementary material

of code words as the feature vector for our polyp classification model. After extensive experimentation with different classification algorithms, we opted for a Support Vector Machine (SVM)[20] with an exponentiated χ^2 kernel as the learning algorithm as suggested by Vempati and colleagues.[21]

Prediction scores of ≥0.33 were deemed good enough for a low-confidence diagnosis. Prediction scores ≥0.50 were deemed good enough for a high-confidence diagnosis. These thresholds were chosen because it is found to produce a high-confidence rate of approximately 83% during the pre-clinical validation. A high-confidence rate of at least 75% was targeted based on levels achieved by previously published studies.[22, 23] After multiple runs of five-fold cross validation the classifier obtained an average diagnostic accuracy level of 84% when the dataset was divided in neoplastic and non-neoplastic lesions.

References

1. Bochkovskiy A, Wang C-Y, Liao H-YM. Yolov4: Optimal speed and accuracy of object detection. arXiv preprint arXiv:2004.10934 2020.
2. Redmon J, Farhadi A. Yolov3: An incremental improvement. arXiv preprint arXiv:1804.02767 2018.
3. Huang G, Liu Z, Van Der Maaten L, et al. Densely connected convolutional networks, In Proceedings of the IEEE conference on computer vision and pattern recognition, 2017.
4. Wang C-Y, Liao H-YM, Wu Y-H, et al. CSPNet: A new backbone that can enhance learning capability of CNN, In Proceedings of the IEEE/CVF conference on computer vision and pattern recognition workshops, 2020.
5. Liu S, Qi L, Qin H, et al. Path aggregation network for instance segmentation, In Proceedings of the IEEE conference on computer vision and pattern recognition, 2018.
6. He K, Zhang X, Ren S, et al. Spatial pyramid pooling in deep convolutional networks for visual recognition. IEEE transactions on pattern analysis and machine intelligence 2015;37:1904-1916.
7. Redmon J, Divvala S, Girshick R, et al. You only look once: Unified, real-time object detection, In Proceedings of the IEEE conference on computer vision and pattern recognition, 2016.
8. Lin T-Y, Maire M, Belongie S, et al. Microsoft coco: Common objects in context, In European conference on computer vision, Springer, 2014.
9. Pan SJ, Yang Q. A survey on transfer learning. IEEE Transactions on knowledge and data engineering 2009;22:1345-1359.
10. He X, Cai D, Niyogi P. Laplacian score for feature selection. Advances in neural information processing systems 2005;18.
11. J.E.G. I, Bastiaansen BA, van Leerdam ME, et al. Development and validation of the WASP classification system for optical diagnosis of adenomas, hyperplastic polyps and sessile serrated adenomas/polyps. Gut 2016;65:963-70.
12. Ojala T, Pietikäinen M, Harwood D. A comparative study of texture measures with classification based on featured distributions. Pattern recognition 1996;29:51-59.
13. Krizhevsky A, Sutskever I, Hinton GE. ImageNet classification with deep convolutional neural networks. Communications of the ACM 2017;60:84-90.
14. Simonyan K, Zisserman A. Very deep convolutional networks for large-scale image recognition. arXiv preprint arXiv:1409.1556 2014.
15. Tamaki T, Sonoyama S, Hirakawa T, et al. Computer-aided colorectal tumor classification in NBI endoscopy using CNN features. arXiv preprint arXiv:1608.06709 2016.
16. Lowe DG. Distinctive image features from scale-invariant keypoints. International journal of computer vision 2004;60:91-110.

Supplementary material

17. Nowak E, Jurie F, Triggs B. Sampling strategies for bag-of-features image classification, In European conference on computer vision, Springer, 2006.
18. Yeung S. Tutorial 2: Image Matching," Stanford AI Lab, [Online]. Available: https://ai.stanford.edu/~syyeung/cvweb/tutorial2.html. [Accessed 18 May 2021].
19. Sculley D. Web-scale k-means clustering, In Proceedings of the 19th international conference on World wide web, 2010.
20. Boser BE, Guyon IM, Vapnik VN. A training algorithm for optimal margin classifiers, In Proceedings of the fifth annual workshop on Computational learning theory, 1992.
21. Vempati S, Vedaldi A, Zisserman A, et al. Generalized RBF feature maps for Efficient Detection, In BMVC, 2010.
22. Rees CJ, Rajasekhar PT, Wilson A, et al. Narrow band imaging optical diagnosis of small colorectal polyps in routine clinical practice: the Detect Inspect Characterise Resect and Discard 2 (DISCARD 2) study. Gut 2017;66:887-895.
23. Vleugels JLA, Dijkgraaf MGW, Hazewinkel Y, et al. Effects of Training and Feedback on Accuracy of Predicting Rectosigmoid Neoplastic Lesions and Selection of Surveillance Intervals by Endoscopists Performing Optical Diagnosis of Diminutive Polyps. Gastroenterology 2018;154:1682-1693.e1.

Fig. 2.1s

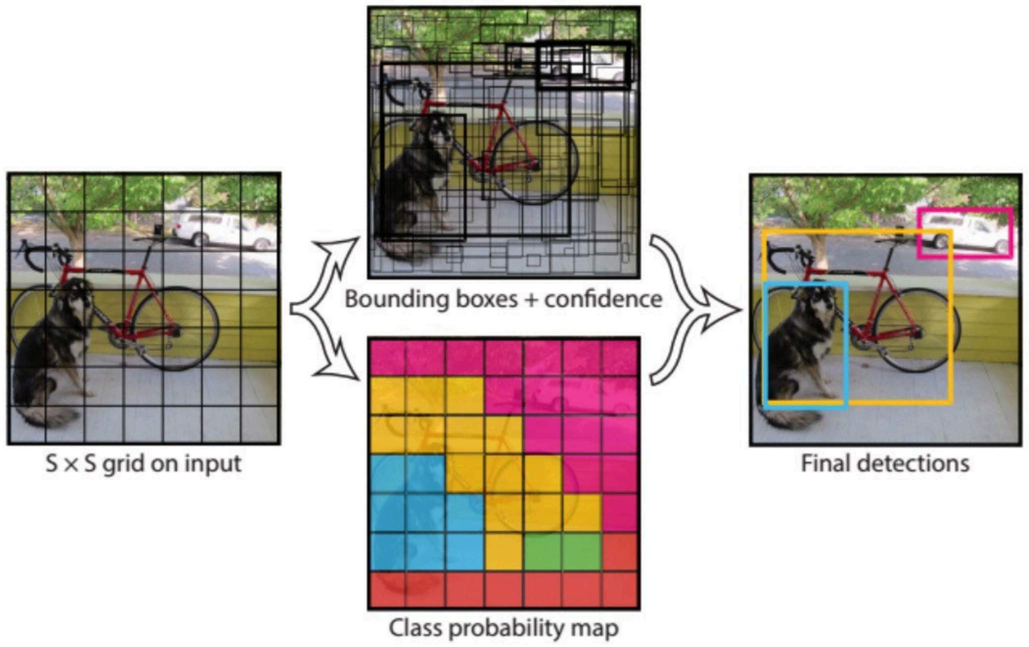

Figure 2: The Model. Our system models detection as a regression problem. It divides the image into an $S \times S$ grid and for each grid cell predicts B bounding boxes, confidence for those boxes, and C class probabilities. These predictions are encoded as an $S \times S \times (B * 5 + C)$ tensor.

Supplementary material

Fig. 2.2s

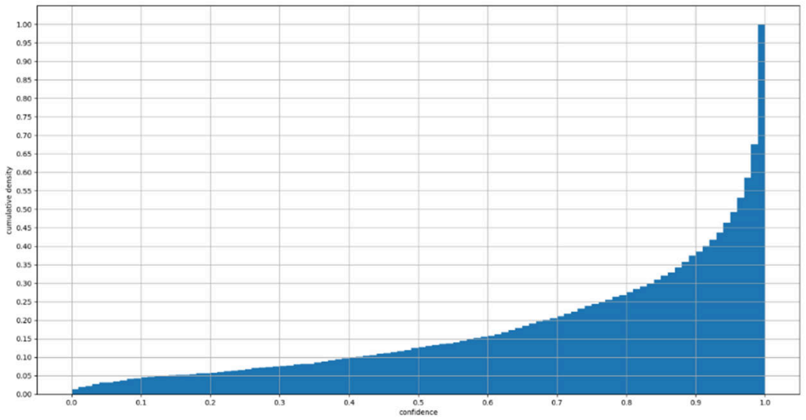

Fig. 2.3s Graphical overview of the polyp classification model

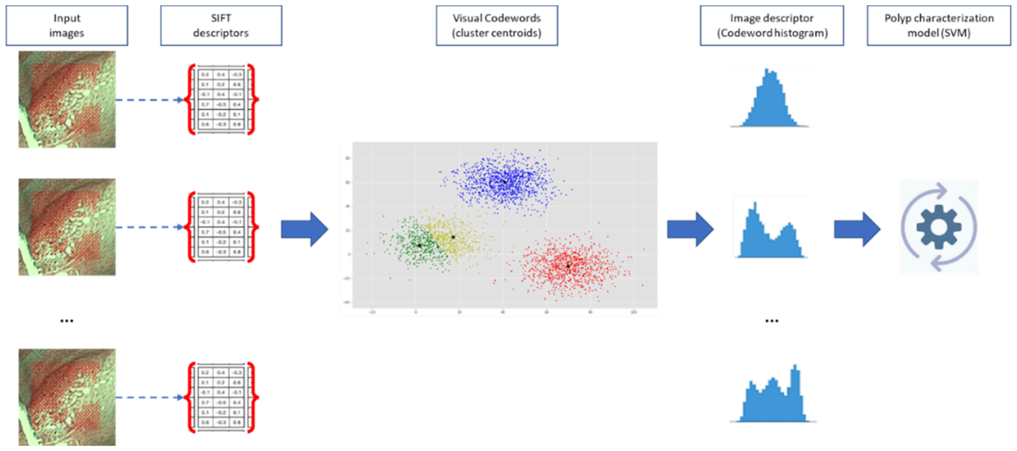

Fig. 2.4s

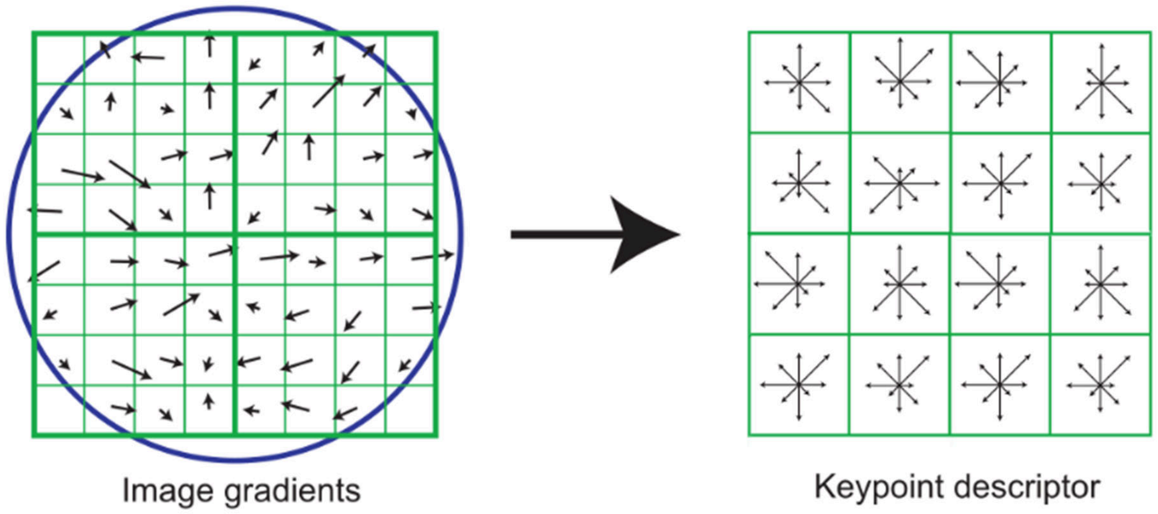

Supplementary material

Fig. 2.5s

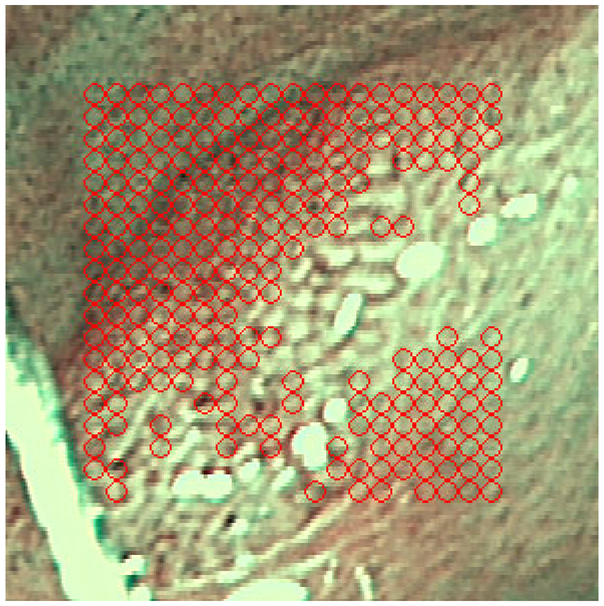

Fig. 2.6s

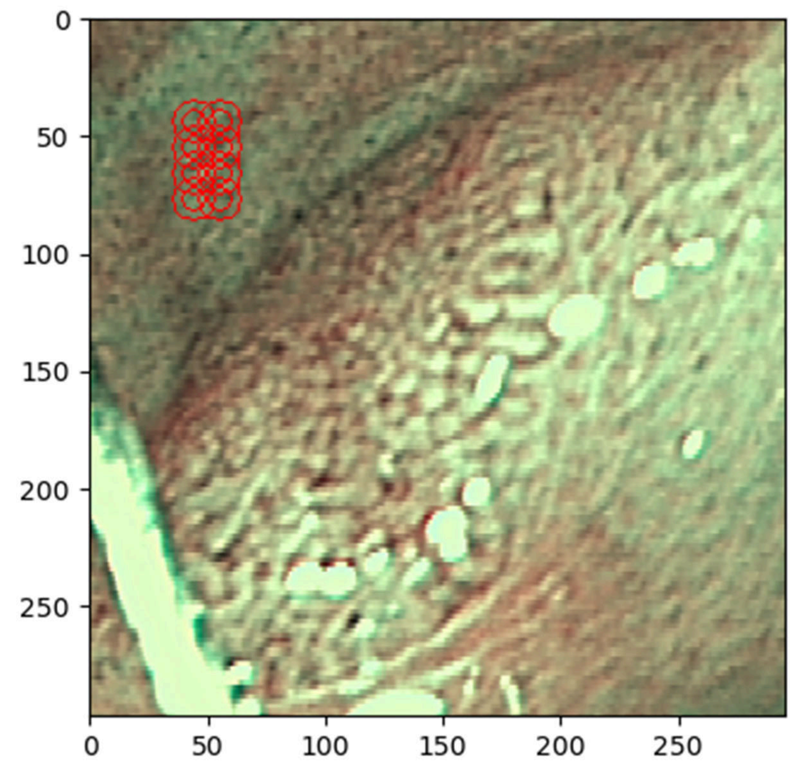

Supplementary material

Appendix 3s Statistical analysis for the negative predictive value (NPV) of neoplastic lesions in the rectosigmoid and agreement of the assigned surveillance intervals

The NPV of neoplastic lesions in the rectosigmoid, based on optical diagnosis of diminutive polyps with high confidence (POLAR system and endoscopists), were compared to histopathology using a two-sided McNemar test. To calculate the NPV for predicting diminutive neoplastic lesions in the rectosigmoid, optical diagnosis of each diminutive polyp with high confidence (POLAR system and endoscopists), was compared with histopathological diagnosis.

The agreement of the assigned surveillance intervals based on optical diagnosis of diminutive colorectal polyps with high confidence were calculated by the number of correct predicted surveillance intervals. The POLAR system and the endoscopists were again compared using the two-sided McNemar test. The reference standard for correct surveillance interval was based on histopathology and the European surveillance guidelines.[24] To examine the impact of optical diagnosis on surveillance interval recommendations, the appropriate recommendation was determined with and without the information of the optical diagnosis (endoscopists and the POLAR system). First, surveillance intervals were determined for each patient combining the results from high-confidence optical diagnosis of diminutive polyps with the pathology diagnosis of all other polyps. Then, surveillance intervals were assigned only based on the actual pathology results and this was used as a reference standard for calculation of the surveillance interval agreement. Patients were excluded from surveillance interval agreement if no diminutive polyps were detected, histopathology outcome was missing, or if only low-confidence predictions were made for diminutive polyps. Patients were also excluded when they were referred for additional treatment of a detected lesion; diagnosed with synchronous CRC, inflammatory bowel disease, or polyposis syndrome; and those in which the surveillance advice was shortened or altered because of other clinical reasons. Patients with incomplete bowel examination (i.e. no cecal intubation or Boston Bowel Preparation Scale <6) were also excluded.

The diagnostic accuracy for optical diagnosis of diminutive polyps if endoscopists are assisted by POLAR-system (AI-assisted optical diagnosis) was calculated as follows. If the endoscopist assessed a diminutive polyp with high confidence, regardless of the optical diagnosis prediction of the POLAR system, the assessment by the endoscopist was used as final optical diagnosis. If the endoscopist assessed a diminutive polyp with low confidence, the optical diagnosis prediction of the POLAR system was only used as final optical diagnosis if it was a high-confidence predictions. If the POLAR system also assessed a diminutive polyp with low confidence, the assessment by the endoscopist was used as final optical diagnosis.

Generalized estimating equations modelling using binary logistic regression adjusted for clustering of polyps and patients per endoscopist was performed to evaluate factors associated with for accurate optical diagnosis by POLAR. The outcome variable of the model was accurate histology prediction of a polyp. Potential predictors included polyp location, size, morphology, confidence level endoscopists, optical diagnosis accuracy of endoscopists, technical characteristics (e.g. localization confidence score, Intersection of Union score, Dice Score, sharpness score, contrast score, overexposed score and classifier confidence score. For the optical diagnosis accuracy of endoscopists the endoscopist were split into the ten endoscopists with the highest accuracy vs the ten endoscopists with lowest accuracy. The association between predictors and accuracy were summarized as odds ratios, including the 95% confidence interval (CI) and corresponding P value. Analysis was performed in statistical software R

Supplementary material

(version 3.6.1) using the EpiR and lme4 packages, with the function of glmer. P-values less than 0.05 were considered statistically significant.

Reference

24. Hassan C, Antonelli G, Dumonceau JM, et al. Post-polypectomy colonoscopy surveillance: European Society of Gastrointestinal Endoscopy (ESGE) Guideline - Update 2020. Endoscopy 2020.

Supplementary material

Table 1s Patient characteristics and colonoscopy quality characteristics

	Patients with a positive FIT (n= 194)
Sex, n (%) male	121 (62%)
Age, median (Q1-Q3 range)	63 (59-70)
ASA classification, n (%)	
1	87 (45%)
2	94 (49%)
3	11(6%)
Missing	2 (0%)
Cecal intubation rate, n (%)	190(98%)
Boston Bowel Preparation Score, median (Q1-Q3 range)	8 (8-9)
Gloucester comfort score, median (Q1-Q3 range)	1 (1-2)
Total withdrawal time in minutes, median (Q1-Q3 range)	15 (10-22)

ASA, American Society of Anesthesiologists; IQR, interquartile range, FIT, fecal immunochemical test

Table 2s Characteristics of the diminutive lesions in the clinical validation set

	Diminutive polyps (N=423)
Location	
Proximal to rectosigmoid	301 (71%)
Rectosigmoid	122 (29%)
Morphology, by Paris classification	
Polypoid (I_p, I_{sp}, I_s)	344 (81%)
Non-polypoid (II_a , II_b, II_c)	77 (19%)
Missing	2 (0%)
Histopathology	
Cancer, n	0
Adenoma, n(%)	300 (71%)
High-grade dysplasia, n	0
≥ 25% villous features, n	5
Sessile serrated lesion, n(%)	41 (10%)
Dysplasia, n	4
Traditional serrated adenoma, n (%)	0 (0%)
Hyperplastic polyp	82(19%)

Supplementary material

Table 3s Diagnostic performance of both CADX system, endoscopists and CADx-assisted for differentiating neoplastic from non-neoplastic diminutive lesions[a]

	Overall			Rectosigmoid		
	CADx system (n=423)	Endoscopists (n=423)	CADx-assisted (n=423)	CADx system (n=122)	Endoscopists (n=122)	CADx-assisted (n=122)
All predictions						
Accuracy (95% CI)	79.4 (75.2-83.2)	83.0 (79.1-86.4)	84.1 (80.2-87.5)	74.6 (65.9-82.0)	84.4 (76.8-90.4)	84.7 (77.0-90.7)
Sensitivity (95% CI)	89.4 (86.2-92.7)	92.4 (89.6-95.2)	93.2 (90.5-95.9)	91.4 (85.4-97.5)	91.7 (85.8-97.6)	90.2 (83.8-96.7)
Specificity (95% CI)	37.8 (27.3-48.3)	43.9 (33.2-54.6)	45.6 (34.6-56.6)	38.8 (22.9-54.8)	68.4 (53.6-83.2)	72.2 (57.6-86.9)
PPV (95% CI)	85.7 (82.0-89.3)	87.3 (83.8-90.7)	88.0 (84.6-91.3)	77.3 (69.0-85.7)	86.5 (79.4-93.6)	88.1 (81.2-95.0)
NPV (95% CI)	46.3 (34.3-58.2)	58.1 (45.8-70.4)	61.0 (48.6-73.5)	66.7 (46.5-86.8)	78.8 (64.8-92.7)	76.5 (62.2-90.7)
	Overall			Rectosigmoid		
	CADx system (n=422)	Endoscopists (n=367)	CADx-assisted (n=415)	CADx system (n=121)	Endoscopists (n=104)	CADx-assisted (n=117)
Only high-confidence predictions						
Accuracy (95% CI)	79.6 (75.5-83.3)	85.8 (81.8-89.2)	84.1 (80.2-87.5)	75.2 (66.5-82.6)	86.9 (79.0-93.7)	84.6 (76.8-90.6)
Sensitivity (95% CI)	89.4 (86.2-92.7)	94.7 (92.1-97.2)	93.2 (90.5-95.9)	91.7 (85.8-97.6)	91.9 (85.7-98.1)	90.2 (83.8-96.7)
Specificity (95% CI)	38.3 (27.7-48.9)	47.1 (35.2-58.9)	44.9 (33.8-55.9)	37.8 (22.2-53.5)	73.3 (57.5-89.2)	71.4 (57.5-86.4)
PPV (95% CI)	85.9 (82.3-89.5)	88.7 (85.2-92.2)	88.8 (84.6-91.3)	77.0 (68.7-85.3)	89.5 (82.6-96.4)	88.1 (81.2-95.0)
NPV (95% CI)	46.3 (34.3-58.2)	66.7 (53.3-80.0)	60.3 (47.8-72.9)	66.7 (46.5-86.8)	78.6 (63.4-93.8)	75.8 (61.1-90.4)

CADx, computer-aided diagnosis; PPV, positive predictive value; NPV, negative predictive value [a]For the calculation of the diagnostic accuracies (neoplastic vs non-neoplastic), adenomas and sessile serrated lesions were considered neoplastic polyps, while hyperplastic polyps were considered non-neoplastic. Note that when an SSL was assessed as an adenoma or vice versa, this was also considered a correct diagnosis because both are categorized as neoplastic lesions.

Supplementary material

Table 4s Outcomes of multilevel logistic regression analysis to find factors associated with accurate optical diagnosis by POLAR

Predictors	Univariate logistic regression Odds Ratio (95% CI)	Multivariate logistic regression Odds Ratio (95% CI)
Polyp Characteristics		
Polyp location		
Rectosigmoid	Reference	Reference
Proximal to rectosigmoid	1.59 (0.92-2.76)	1.54 (0.87-2.72)
Polyp size, mm		
1-2	Reference	Reference
3-5	0.55 (0.32-0.95)	0.48(0.27-0.85)
Morphology		
Polypoid[a]	Reference	Reference
Non-polypoid[b]	0.37 (0.21-0.66)	0.45 (0.27-0.85)
Colonoscopy Characteristics		
Optical diagnosis accuracy endoscopists		
ten endoscopists with lowest accuracy	Reference	Reference
ten endoscopists with highest accuracy	2.25 (1.30-3.89)	1.76 (1.01-3.17)
Confidence level		
Low-confidence	Reference	
High-confidence	1.72 (0.83-3.55)	
Technical Characteristics		
Localization confidence score		
<0.9	Reference	
≥0.9	1.29 (0.78-2.14)	
Intersection over Union score		
<0.8	Reference	
≥0.8	1.45 (0.88-2.39)	
Dice coefficient score		
<0.9	Reference	
≥0.9	1.50 (0.91-2.48)	
Sharpness score		
<300	Reference	
≥300	1.21 (0.72-2.06)	
Contrast score		
<30	Reference	
≥30	1.04 (0.63-1.72)	
Overexposed score		
≥ 2	Reference	Reference
< 2	0.50 (0.30-0.84)	0.63 (0.36-1.07)
Classifier confidence score		
<0.80	Reference	Reference
≥0.80	5.34 (3.16-9.02)	5.74 (3.34-10.13)

[a]Polypoid was defined as Paris classification Ip, Isp and Is; [b] Non-polypoid was defined as Paris classification IIa and IIb

Supplementary material

Table 5s Diagnostic performance of CADx system and endoscopists in differentiating diminutive colorectal polyps (adenomas vs hyperplastic polyps)[a]

	Overall		Proximal to rectosigmoid		Rectosigmoid	
	CADx system (n=334)	Endoscopists (n=334)	CADx system (n=232)	Endoscopists (n=232)	CADx system (n=102)	Endoscopists (n=102)
All predictions						
Accuracy (95% CI)	83.5 (79.1-87.4)	87.4 (83.4-90.8)	85.3 (80.1-89.6)	86.6 (81.6-90.7)	79.4 (70.3-86.8)	89.2 (81.5-94.5)
Sensitivity (95% CI)	93.2 (90.2-96.2)	92.8 (89.8-95.9)	92.2 (88.6-95.9)	92.2 (88.6-95.9)	95.8 (91.2-100)	94.4 (89.2-99.7)
Specificity (95% CI)	34.6 (22.0-47.1)	60.0 (47.1-73.0)	28.0 (10.4-45.6)	40.0 (20.8-59.2)	40.0 (23.5-57.5)	76.7 (61.5-91.8)
PPV (95% CI)	87.8 (84.1-91.6)	92.2 (89.0-95.3)	91.4 (87.6-95.2)	92.7 (89.2-96.3)	79.3 (70.8-87.8)	90.7 (84.1-97.3)
NPV (95% CI)	50.0 (34.1-65.9)	62.2 (49.2-75.3)	30.4 (11.6-49.2)	38.5 (19.8-57.2)	80.0 (59.8-100.0)	85.2 (71.8-98.6)
	Overall		Proximal to rectosigmoid		Rectosigmoid	
	CADx system (n=333)	Endoscopists (n=297)	CADx system (n=232)	Endoscopists (n=206)	CADx system (n=101)	Endoscopists (n=91)
Only high-confidence predictions						
Accuracy (95% CI)	83.8 (79.4-87.6)	89.6 (85.5-92.8)	85.3 (80.1-89.6)	89.3 (84.2-93.1)	80.2 (71.1-87.5)	90.2 (82.2-95.4)
Sensitivity (95% CI)	93.2 (90.2-96.2)	94.8 (92.0-97.5)	92.9 (89.1-96.7)	95.1 (92.0-98.2)	95.8 (91.2-1000	94.0 (88.4-99.7)
Specificity (95% CI)	35.2 (22.5-47.9)	62.5 (48.8-76.2)	30.4 (11.6-49.2)	43.5 (23.2-63.7)	41.4 (23.5-59.3)	80.0 (64.3-95.7)
PPV (95% CI)	88.1 (84.4-91.8)	93.0 (89.8-96.1)	91.4 (87.3-95.4)	93.0 (89.4-96.7)	80.2 (71.8-88.7)	92.7 (86.4-98.9)
NPV (95% CI)	50.0 (34.1-65.9)	69.8 (56.0-83.5)	35.0 (14.1-55.9)	52.6 (30.2-75.1)	80.0 (59.8-100)	83.3 (68.4-98.2)

CAD, computer-aided diagnosis; PPV, positive predictive value; NPV, negative predictive value. [a]Histologically and endoscopically diagnosed sessile serrated lesions are excluded for these analyses
